# Editors’ Book Table

**Published:** 1871-02

**Authors:** 


### Editor's Book Table.

[NOTE.—All works reviewed in the columns of the CHICAGO MEDICAL JOURNAL may be found in the extensive stock of W. B. KEEN & COOKE, whose catalogue of Medical Books will be sent to any address upon request.]

*Lectures upon Diseases of the Rectum.* Delivered at the Bellevue Hospital Medical College, Session 1869–70, by W. H. VAN BUREN, A.M., M.D., etc., etc. New York: D. Appleton & Co. 1870. Pp. 164.

This little volume is composed of eight lectures, which the author tells us "have been written out for publication in deference to the wishes of a number of, perhaps, too partial friends."

Dr. Van Buren's large experience in this class of diseases enables him to speak authoritatively; and we have perused the present work with interest. But we confess to surprise that Prof. Van Buren should have suffered himself to fall into an anatomical error by following too closely upon traditional pathology. The poor old liver has ever been a sort of scape-goat, general to all classes of physicians; and surgeons have always attributed to obstructed portal circulation too large an influence in the production of piles. Following in this lead, our author not only recognizes this obstruction as a cause of hæmorrhoids, but attempts to account for it by a piece of erroneous anatomy. He says:

"There is a net-work of good-sized veins surrounding the lower end of the rectum for an inch or two, in the rather abundant connective tissue between its mucous membrane and the layer of circular muscular fibres surrounding it, which is known as the 'hæmorrhoidal plexus.' *These empty into the inferior mesenteric vein*," etc.

We have italicised the last sentence because it contains the error to which we have alluded. The hæmorrhoidal veins are three in number, and one of them only—the superior—flows into the mesenteric. The other two—the middle and inferior—empty into the internal iliac. Consequently, obstructed portal circulation cannot exert so potent an influence in producing piles as it would were Prof. Van Buren's account of the anatomy of the veins correct. With two-thirds of the flow through an unobstructed outlet, and that two-thirds proceeding directly from that portion of the hæmorrhoidal plexus which is the seat of piles, both external and internal, obstruction of the other third cannot exert so great an influence as Prof. Van Buren, in common with most authors and teachers, attributes to it. It is only another instance of the influence of tradition upon our teaching and practice.

With this single exception, we can speak of the book in terms of unqualified praise; and as this exception does not necessarily involve a great variation in practice, it is of consequence only as an actual error which should be avoided. We recommend the book to general practitioners, for we are confident they will find hints which they may follow to advantage, and which will enable them to manage diseases of the rectum with more satisfaction than sometimes attends their practice.

